# Supplementary material for: demuxmix: demultiplexing oligonucleotide-barcoded single-cell RNA sequencing data with regression mixture models
Source: Bioinformatics. 2023 Aug 1;39(8):btad481. doi: 10.1093/bioinformatics/btad481 (PMC10412409; doi:10.1093/bioinformatics/btad481)
Supplement: btad481_Supplementary_Data [file btad481_supplementary_data.pdf]

Supplementary Information

demuxmix: Demultiplexing oligonucleotide-barcoded single-cell RNA sequencing data with regression mixture models

1. Supplementary Methods Page 1

2. Supplementary Tables S1 and S2 Page 4

3. Supplementary Figures S1 to S5 Page 5

4. Supplementary R code Page 10

1. Supplementary methods

1.1 Performance metrics

Demultiplexing is generally a multiclass problem, and not all classification errors have an equally detrimental impact on subsequent analyses. Choosing an appropriate performance metric is further complicated by the use of additional classes, such as 'uncertain' or 'negative', which demultipexing methods assign to droplets with ambiguous or weak HTO signals. Typically, only single-sample droplets (SSDs) are retained for downstream analyses, making the accurate classification of SSDs particularly important. Here, the $\text{precision}_{\text{SSD}}$ was used as performance metric, which was defined as the probability that a classification is correct given that the droplet was assigned to any SSD class:

$$\text{precision}_{\text{SSD}} = P(\hat{c}_i = C_i | \hat{c}_i \in \text{SSD})$$

The $\text{precision}_{\text{SSD}}$ is calculated solely based on droplets that were assigned to an SSD class. It does not account for true SSDs that might have been incorrectly classified as multi-sample multiplets (MSMs) or assigned to the 'uncertain' class. To assess the methods' ability to detect SSDs, the $\text{sensitivity}_{\text{SSD}}$ was employed. It was defined as the probability of assigning a droplet to any SSD class given that the droplet is indeed a true SSD:

$$\text{sensitivity}_{\text{SSD}} = P(\hat{c}_i \in \text{SSD} | C_i \in \text{SSD})$$

These two performance measures are complimentary. A low $\text{precision}_{\text{SSD}}$ indicates that there will be a high number of incorrect assignments used in downstream analyses, potentially leading to false conclusions. A low $\text{sensitivity}_{\text{SSD}}$ means that useable data from true SSDs is being discarded, which can negatively impact the power of downstream analyses. To combine the precision and sensitivity of a classifier into a single metric, the F-score was used. Specifically, F_{SSD} was defined as the harmonic mean of $\text{precision}_{\text{SSD}}$ and $\text{sensitivity}_{\text{SSD}}$:

$$F_{\text{SSD}} = 2 / (\text{precision}_{\text{SSD}}^{-1} + \text{sensitivity}_{\text{SSD}}^{-1})$$

The F_{SSD} is reported together with the $\text{precision}_{\text{SSD}}$ and $\text{sensitivity}_{\text{SSD}}$ in Tables 1 and 2 in the main manuscript.

The first three metrics focus on the recovery of SSDs and the correct classification of SSDs. While MSMs are usually not used in the final data analyses, it can still be helpful to correctly classify them instead of assigning them to the 'uncertain' or 'negative' class. For example, if MSMs form a distinct cluster together with SSDs, it may indicate that those SSDs are single-sample multiplets, which are challenging to detect. Additionally, obtaining an accurate estimation of the MSM rate can aid in optimizing the experimental design. Two measures were computed to evaluate the correct classification of MSMs. The $\text{precision}_{\text{MSM}}$ is defined similarly to the $\text{precision}_{\text{SSD}}$:

$$\text{precision}_{\text{MSM}} = P(C_i \in \text{MSM} | \hat{c}_i \in \text{MSM})$$

The $\text{precision}_{\text{MSM}}$ is the probability that a predicted MSM is indeed a true MSM, with the exact MSM class being disregarded for simplicity.

Additionally, the predicted MSM rate was calculated by ignoring all droplets classified as 'uncertain' or 'negative':

$$\text{MSM rate} = |\{\hat{c}_i : \hat{c}_i \in \text{MSM}\}| \,/\, |\{\hat{c}_i : \hat{c}_i \in \text{SSD} \cup \text{MSM}\}|$$

Supplementary Tables S1 and S2 provide the observed $\text{precision}_{\text{MSM}}$ and the MSM rate error for the evaluated methods. The MSM rate error was calculated as the difference between the predicted MSM rate and the true MSM rate of the simulated dataset.

Another commonly used metric for evaluating classifiers is the Matthews correlation coefficient (MCC), which can be adapted for multiclass problems (Gorodkin, 2004). To calculate the MCC, the MSM classes were combined into a single MSM class. The classes 'uncertain' and/or 'negative' were also combined into one class, resulting in a total of n+2 classes.

To calculate the MCC, the n-dimensional vector $\hat{c}_i$ encoding the predicted class of droplet i was rewritten as a vector $\hat{d}_i \in \{0,1\}^{n+2}$, where one and only one element equals 1 indicating the predicted class of droplet i. The $m \times (n+2)$ matrix $\widehat{K}$ encodes the predicted classes for all m droplets, and K is the respective $m \times (n+2)$ matrix encoding the true classes. The MCC was calculated as:

$$\text{MCC} = \frac{cov(\widehat{K},K)}{\sqrt{cov(\widehat{K},\widehat{K})\, cov(K,K)}} \quad \text{with} \quad cov(\widehat{K},K) = \frac{1}{n+2}\sum_{j=1}^{n+2} cov(\widehat{K}_{\cdot,j}, K_{\cdot,j})$$

The MCC takes SSDs and MSMs into account and treats all classification errors equally. However, it penalizes methods that assign many droplets to the 'uncertain' class since any droplet classified as 'uncertain' is counted as an error. This is disadvantageous for conservative methods that prioritize avoiding falsely classified SSDs. The MCC values can be found in Supplementary Tables S1 and S2.

1.2 Parameter settings and software versions used in the benchmark study

Default parameters were used for all methods in the benchmark study. The demuxmix method from the R/Bioconductor package (version 1.0.0) was used with default settings, including the parameter pAcpt = 0.9^n, where n represents the number of HTOs in the dataset. The parameter model was set to 'auto'. Raw HTO counts were used as input, and the number of detected genes was defined as the number of features in the RNA library with at least one read. The same settings were used for the demuxmix naïve method, except that the parameter model was set to 'naive'.

For MULTI-seq, the MULTIseqDemux() function from the R package Seurat (version 4.1.1) was employed. The autoThresh parameter was set to TRUE. All other parameters remained at their default values. As recommended in the MULTI-seq documentation, HTO counts were normalized prior to running MULTI-seq using the centered log ratio transformation (CLR) implemented in Seurat's NormalizeData() function.

HTODemux, provided by the R package Seurat (version 4.1.1), was used with default settings, including the 0.99 quantile as the threshold for defining positive cells (positive.quantile = 0.99). As recommended, the same CLR normalization as for MULTI-seq was applied before running HTODemux.

The Python package demuxEM (version 0.1.7) was employed with default parameters and a min-num-genes value of 200. demuxEM required the full HTO datasets including empty droplets to estimate the background HTO distribution.

The hashedDrops implementation from the Bioconductor package DropletUtils (version 1.16.0) was used with default settings. As per default, the parameter ambient was set to NULL, and doublet.mixture was set to FALSE. Raw HTO counts after filtering out empty droplets were used as input.

The Python package GMM-Demux (version 0.2.1.3) was applied without setting or changing any optional parameters, meaning that the default confidence threshold of 0.8 was used. Raw HTO counts were provided as input.

1.3 Clustering of the cell line mixture dataset

In the cell line mixture dataset, the distinct transcription profiles of the cell lines were used to demultiplex the droplets independently of the HTO data. A standard single-cell clustering workflow described by Amezquita, et al. (2020) was applied. Empty droplets were removed using the emptyDrops() method (Lun, et al., 2019). The remaining 7,596 droplets were normalized and log-transformed using the logNormCounts() method from the R package scater. The top 5,000 genes with the largest biological component of variance were selected using modelGeneVar(). Principal component analysis (PCA) was then applied to the top 500 most variable genes from this set. Droplets were clustered using the Walktrap community detection algorithm applied to the n=10 nearest-neighbor graph constructed from the top 50 principle components (Pons and Latapy, 2006). A total of 19 clusters were detected. The larger clusters (≥ 200 droplets) represented the different cell lines, and droplets from these clusters were labeled accordingly. However, one cluster of 294 cells showed a high amount of mitochondrial reads (mean of 16.6%), indicating apoptotic cells. This

cluster, along with the remaining smaller clusters that likely reflected multiplets, were labeled as ‘uncertain’. The R code used to preprocess and cluster the dataset is attached to this document.

References

Amezquita, R.A., *et al.* Orchestrating single-cell analysis with Bioconductor. *Nat Methods* 2020;17(2):137-145.

Gorodkin, J. Comparing two K-category assignments by a K-category correlation coefficient. *Comput Biol Chem* 2004;28(5-6):367-374.

Lun, A.T.L., *et al.* EmptyDrops: distinguishing cells from empty droplets in droplet-based single-cell RNA sequencing data. *Genome Biol* 2019;20(1):63.

Pons, P. and Latapy, M. Computing communities in large networks using random walks. *Journal of Graph Algorithms and Applications* 2006;10(2):192-218.

2. Supplementary tables

Supplementary Table S1. Additional scores from the simulation based on the human brain dataset averaged over the results obtained by applying scaling factors ranging from 1 to 0.1.

Method	$Precison_{MSM}$	MSM rate error	MCC
demuxmix	0.8481 (#2)	0.0146 (#3)	0.9287 (#1)
$demuxmix^{naive}$	0.8015 (#5)	0.0193 (#4)	0.9226 (#3)
MULTI-seq	0.8261 (#4)	0.0213 (#5)	0.9253 (#2)
HTODemux	0.7204 (#6)	0.0373 (#6)	0.9138 (#4)
DemuxEM	0.5999 (#7)	0.0650 (#7)	0.9003 (#5)
hashedDrops	0.8728 (#1)	-0.0109 (#2)	0.8656 (#6)
GMM-Demux	0.8467 (#3)	-0.0043 (#1)	0.8634 (#7)

MSM rate error was defined as the estimated MSM rate minus the true MSM rate.

Supplementary Table S2. Additional scores from the simulation based on the cell line mixture dataset averaged over the results obtained by applying scaling factors ranging from 1 to 0.25.

Method	$Precison_{MSM}$	MSM rate error	MCC
demuxmix	0.3347 (#3)	0.1003 (#4)	0.8277 (#1)
$demuxmix^{naive}$	0.3339 (#4)	0.1000 (#3)	0.8270 (#2)
MULTI-seq	0.4637 (#1)	0.0563 (#2)	0.7858 (#3)
HTODemux	0.2315 (#5)	0.1719 (#5)	0.7497 (#4)
DemuxEM	0.1797 (#6)	0.2099 (#6)	0.7256 (#5)
hashedDrops	0.3964 (#2)	0.0092 (#1)	0.6477 (#6)
GMM-Demux	N/A	N/A	N/A

MSM rate error was defined as the estimated MSM rate minus the true MSM rate.
GMM-Demux was excluded since it did not detect SSDs for $s < 0.5$.

3. Supplementary Figures

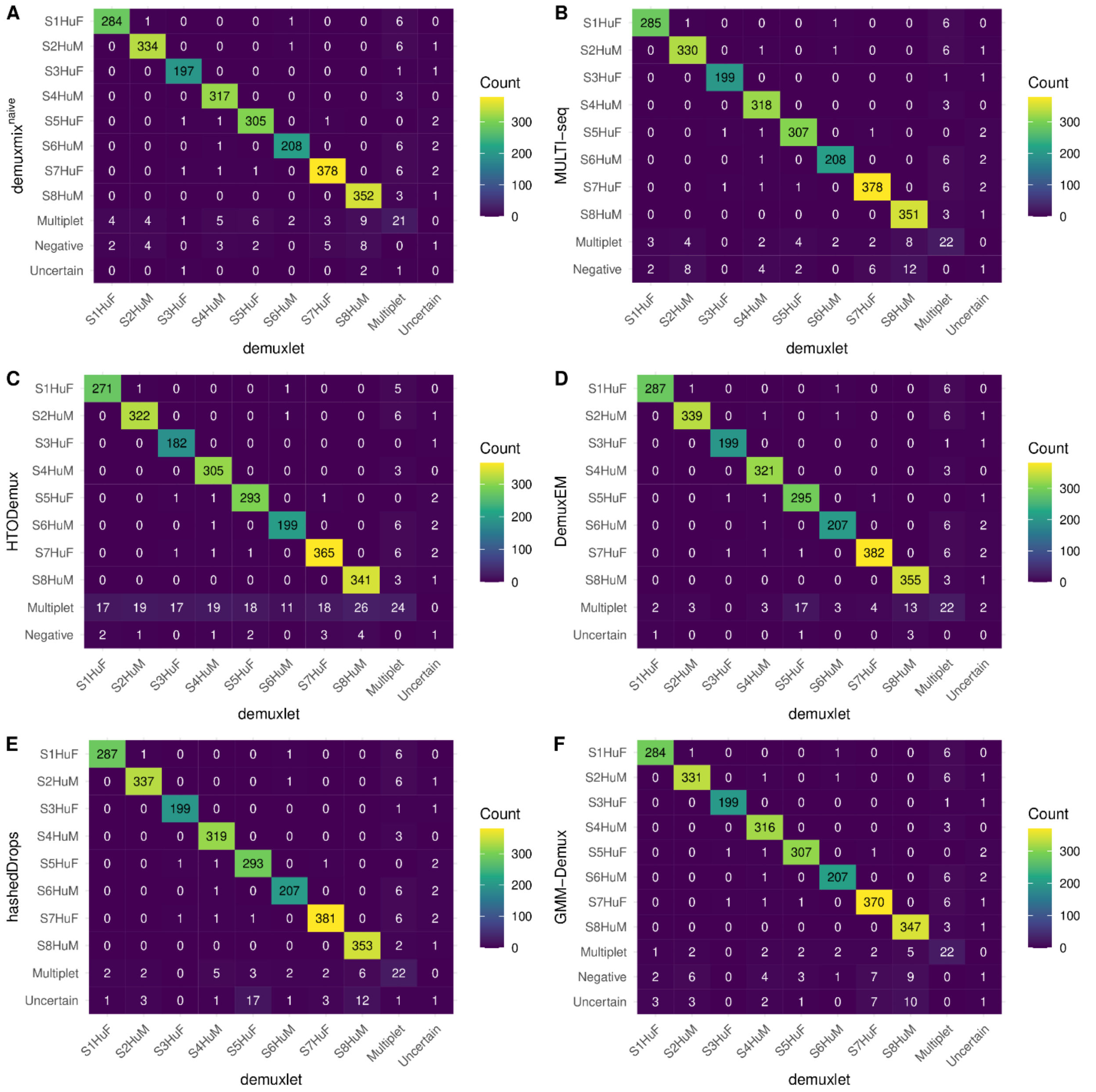

Supplementary Fig. S1. Concordance between HTO-based demultiplexing and genetic demultiplexing for the human brain dataset. A-F) Confusion matrix shows the demultiplexing results from demuxlet (genetic demultiplexing) on the x-axis versus the results obtained from **A)** demuxmix naïve, **B)** MULTI-seq, **C)** HTODemux, **D)** DemuxEM, **E)** hashedDrops, and **F)** GMM-Demux on the y-axis. Results are shown for all 2,509 droplets in the original dataset. The respective confusion matrix for demuxmix is shown in Fig. 1E in the main manuscript.

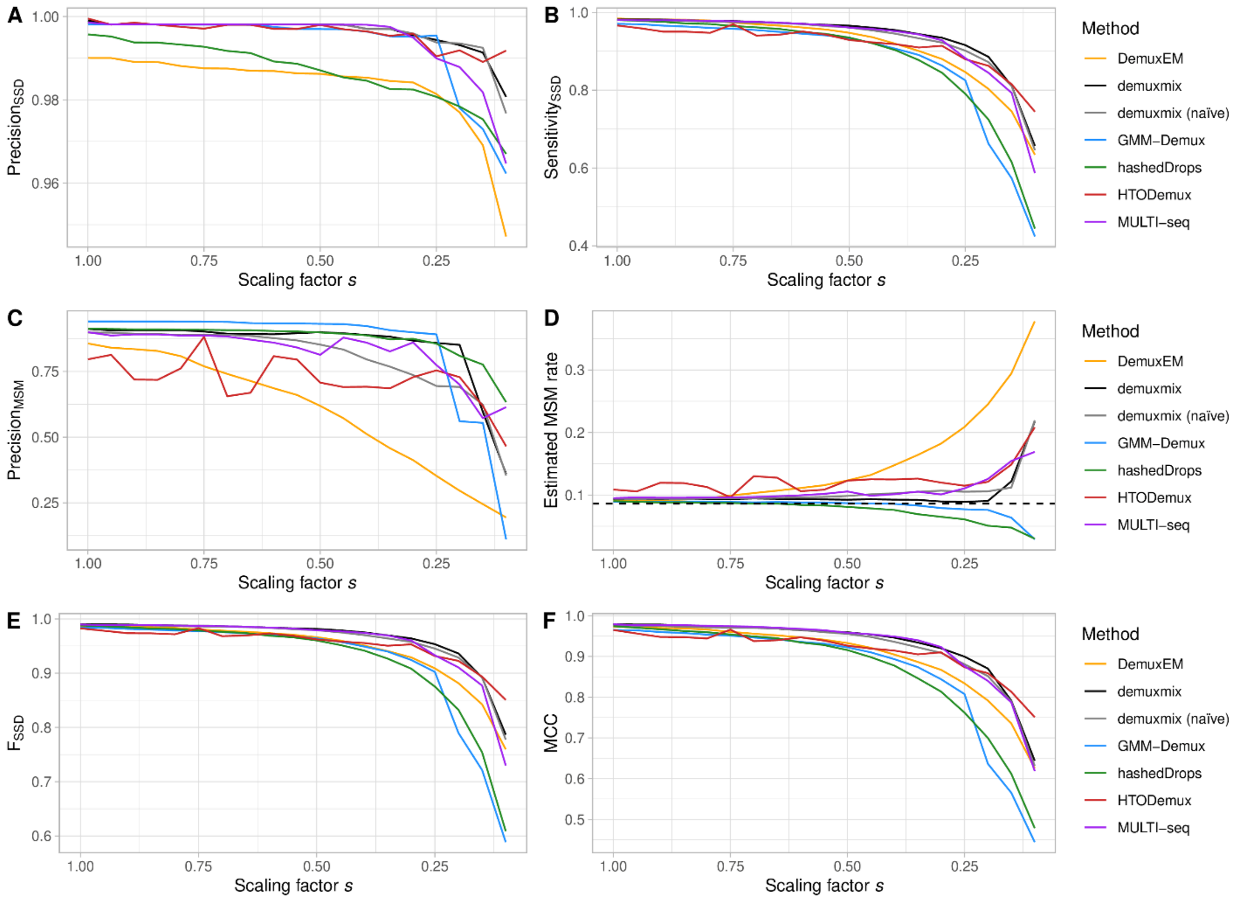

Supplementary Fig. S2. Benchmark results from the human brain dataset. A) Plot shows the precision$_{\mathrm{SSD}}$ on the y-axis for different scaling factors *s* on the x-axis. The read counts of the HTO used to tag the cell but not the background HTO counts were multiplied with *s* to reduce the HTO signal. Precision$_{\mathrm{SSD}}$ was defined as the probability that the classification is correct given an SSD class was predicted: $P(\hat{c}_i = C_i | \hat{c}_i \in \mathrm{SSD})$. **B)** Plot shows the sensitivity$_{\mathrm{SSD}}$ on the y-axis for different scaling factors *s* on the x-axis. Sensitivity$_{\mathrm{SSD}}$ was defined as the probability that a true SSD was assigned to any SSD class: $P(\hat{c}_i \in \mathrm{SSD} | C_i \in \mathrm{SSD})$. **C)** Plot shows the precision$_{\mathrm{MSM}}$ on the y-axis for different scaling factors *s* on the x-axis. Precision$_{\mathrm{MSM}}$ was defined as the probability that a predicted MSM is a true MSM: $P(C_i \in \mathrm{MSM} | \hat{c}_i \in \mathrm{MSM})$. **D)** Plot shows the estimated MSM rate on the y-axis for different scaling factors *s* on the x-axis. The true MSM rate in the simulated data is shown as black dashed line. **E)** Plot shows the F-score on the y-axis for different scaling factors *s* on the x-axis. The F-score was defined as the harmonic mean of the precision$_{\mathrm{SSD}}$ and the sensitivity$_{\mathrm{SSD}}$. **F)** Plot shows the Matthews Correlation Coefficient (MCC) on the y-axis for different scaling factors *s* on the x-axis.

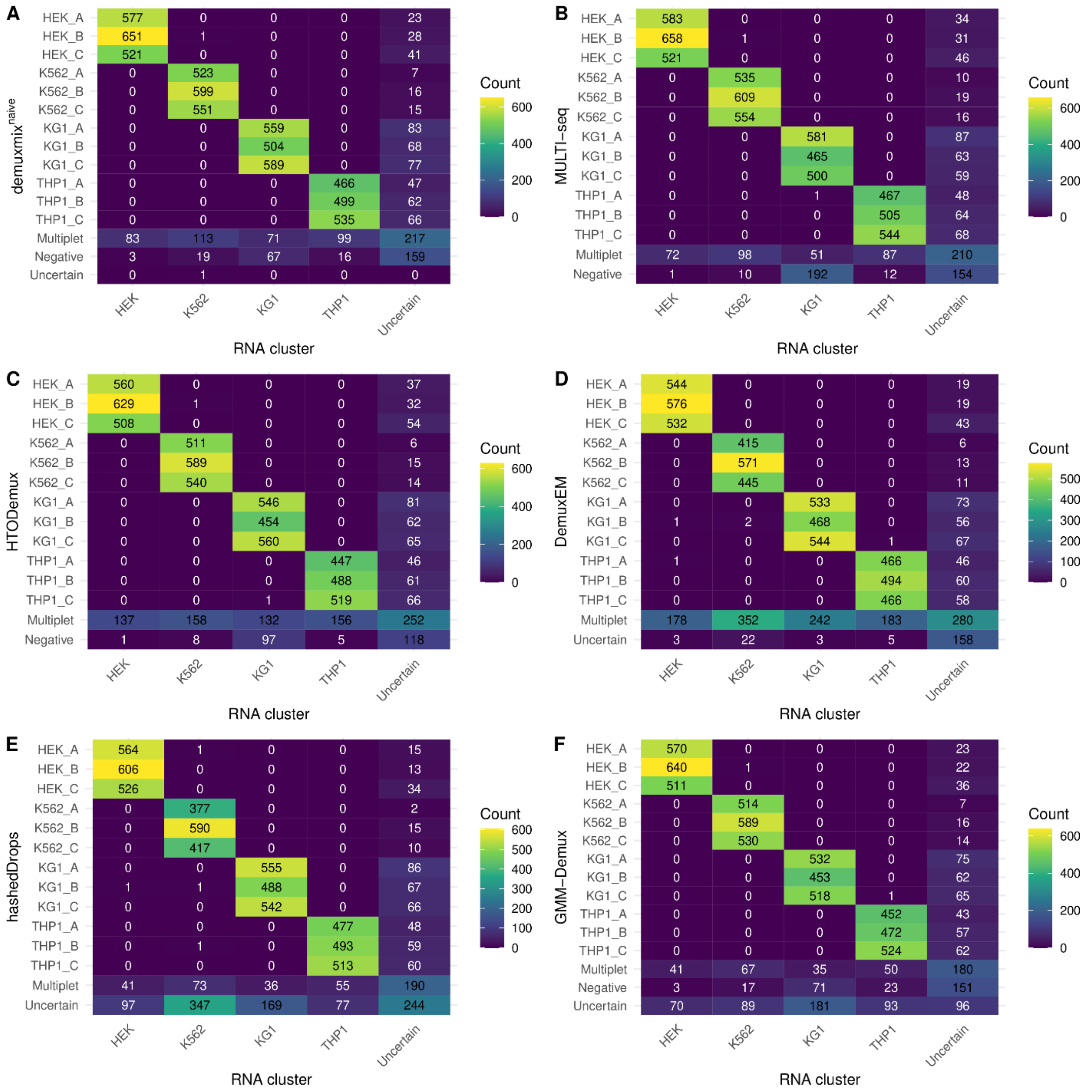

Supplementary Fig. S3. Concordance between HTO-based demultiplexing and transcriptomic clustering for the cell line mixture dataset. A-F) Confusion matrix shows the cell identities derived from transcriptomic cell clustering on the x-axis versus the results obtained from **A)** demuxmix naïve, **B)** MULTI-seq, **C)** HTODemux, **D)** DemuxEM, **E)** hashedDrops, and **F)** GMM-Demux on the y-axis. Results are shown for all 7,956 droplets in the original dataset. The respective confusion matrix for demuxmix is shown in Fig. 2E in the main manuscript.

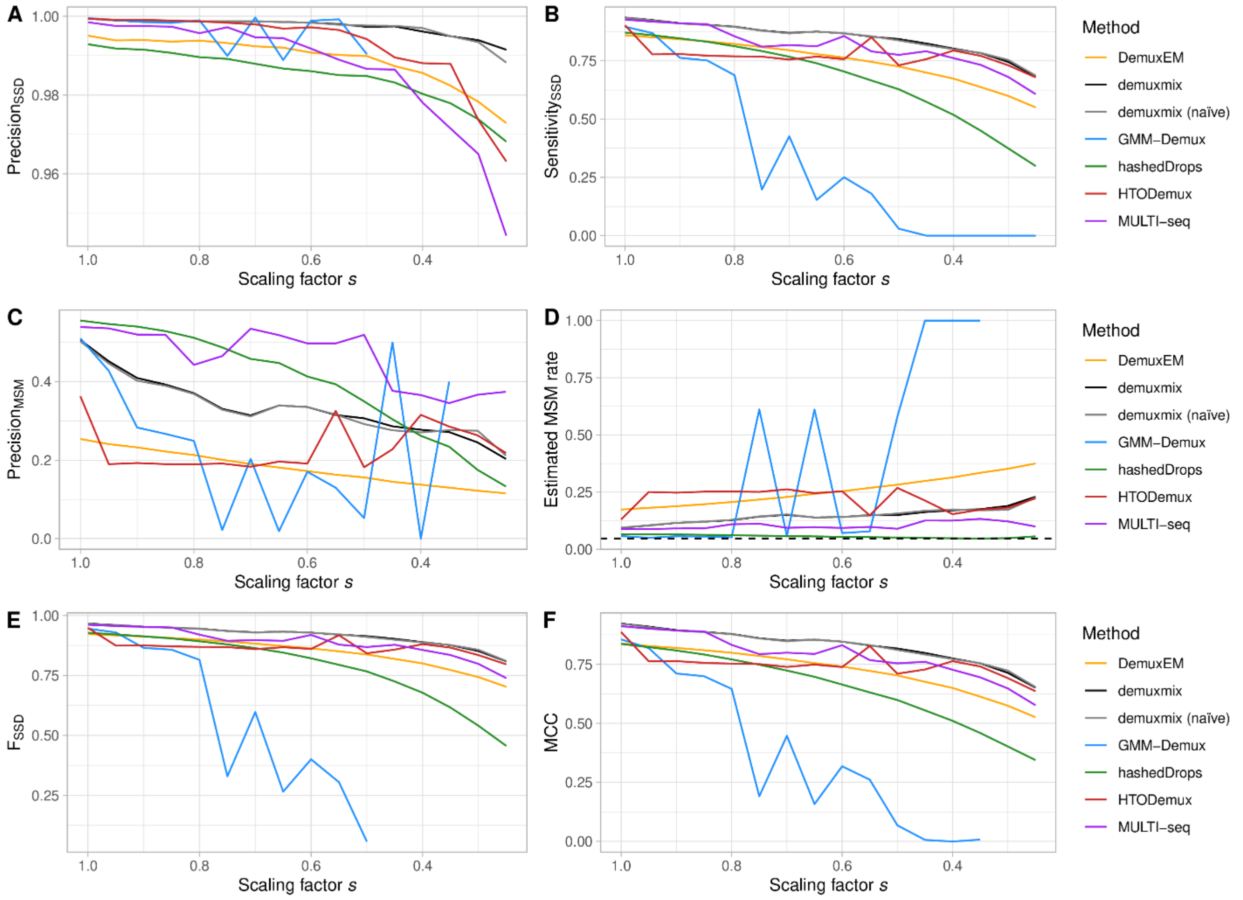

Supplementary Fig. S4. Benchmark results from the cell line mixture dataset. A) Plot shows the precision$_{SSD}$ on the y-axis for different scaling factors s on the x-axis. The read counts of the HTO used to tag the cell but not the background HTO counts were multiplied with s to reduce the HTO signal. Precision$_{SSD}$ was defined as the probability that the classification is correct given an SSD class was predicted: $P(\hat{c}_i = C_i | \hat{c}_i \in \mathrm{SSD})$. **B)** Plot shows the sensitivity$_{SSD}$ on the y-axis for different scaling factors s on the x-axis. Sensitivity$_{SSD}$ was defined as the probability that a true SSD was assigned to any SSD class: $P(\hat{c}_i \in \mathrm{SSD} | C_i \in \mathrm{SSD})$. **C)** Plot shows the precision$_{MSM}$ on the y-axis for different scaling factors s on the x-axis. Precision$_{MSM}$ was defined as the probability that a predicted MSM is a true MSM: $P(C_i \in \mathrm{MSM} | \hat{c}_i \in \mathrm{MSM})$. **D)** Plot shows the estimated MSM rate on the y-axis for different scaling factors s on the x-axis. The true MSM rate in the simulated data is shown as black dashed line. **E)** Plot shows the F-score on the y-axis for different scaling factors s on the x-axis. The F-score was defined as the harmonic mean of the precision$_{SSD}$ and the sensitivity$_{SSD}$. **F)** Plot shows the Matthews Correlation Coefficient (MCC) on the y-axis for different scaling factors s on the x-axis.

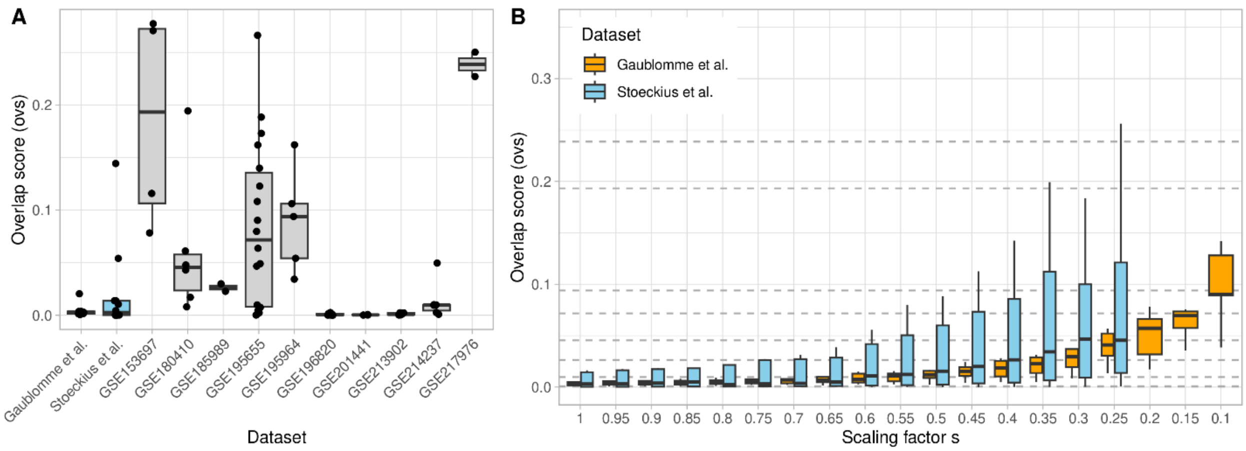

Supplementary Fig. S5. Quality assessment of published HTO datasets. A) The boxplot illustrates the distribution of the overlap score (ovs) on the y-axis for the human brain dataset (Gaublomme et al., depicted in orange), the cell line mixture dataset (Stoeckius et al., depicted in light blue), and ten recently published HTO datasets. Each dot represents one HTO used in the respective study. The first ten datasets retrieved from a Gene Expression Omnibus query for 'hashtag oligonucleotides' that met the following criteria were selected: i) generated from human samples, ii) published in a peer-reviewed journal, and iii) HTO counts for all droplets available. **B)** The boxplot presents the overlap score for the human brain dataset (Gaublomme et al., depicted in orange) and the cell line mixture dataset (Stoeckius et al., depicted in light blue) for different scaling factors *s* used in the simulation to dilute the HTO signal. Dots representing the ovs of individual HTOs were omitted for clarity. Each of the gray dashed lines corresponds to a median overlap score observed in one of the ten published datasets shown in panel A).

4. Supplementary R code

The following R code was used to cluster and assign cells to cell lines based on their RNA profiles. The assignment was used as ground truth for the cell line mixture dataset.

```
library(DropletUtils)
library(scran)
library(scater)

# Read RNA count data
# [ftp.ncbi.nlm.nih.gov/geo/samples/GSM3501nnn/GSM3501446/suppl/GSM3501446_MixCellLines-RNA.umi.txt.gz]
counts <- read.csv("GSM3501446_MixCellLines-RNA.umi.txt", sep="\t", header=TRUE)
counts <- as.matrix(counts)

# Read HTO count data
# [ftp.ncbi.nlm.nih.gov/geo/samples/GSM3501nnn/GSM3501447/suppl/GSM3501447_MixCellLines-HTO-counts.csv.gz]
htos <- read.csv("GSM3501447_MixCellLines-HTO-counts.csv", header=TRUE)
rownames(htos) <- htos$X
htos$X <- NULL
htos$no_match <- NULL
htos$ambiguous <- NULL
htos$total_reads <- NULL
htos$bad_struct <- NULL
htos <- t(as.matrix(htos))

# Create SingleCellExperiment object
shared <- colnames(counts)[colnames(counts) %in% colnames(htos)]
sce <- SingleCellExperiment(
    assays=SimpleList(counts=counts[, shared]),
    altExps=list(hto=SummarizedExperiment(assays=SimpleList(counts=htos[, shared])))
)

# Remove empty droplets
set.seed(10000)
e.out.gene <- emptyDrops(counts(sce))
is.cell <- e.out.gene$FDR <= 0.001
summary(is.cell)
#    Mode   FALSE    TRUE    NA's
# logical    1362    7956   15770
sce <- sce[,which(is.cell)]

# Add QC metrics
mtGenes <- grep("MT-", rownames(sce))
sce <- addPerCellQC(sce, subsets=list(Mito=mtGenes))

# Normalize data, run PCA + TSNE
sce <- logNormCounts(sce)
dec <- modelGeneVar(sce)
set.seed(100)
sce <- runPCA(sce, subset_row=getTopHVGs(dec, n=5000))
sce <- runTSNE(sce, dimred="PCA")

# Cluster cells
g <- buildSNNGraph(sce, k=10, use.dimred="PCA")
cluster <- igraph::cluster_walktrap(g)$membership
sce$cluster <- factor(cluster)
plotTSNE(sce, colour_by="cluster")
boxplot(sce$subsets_Mito_percent ~ sce$cluster) # cluster 2 has KG1 cells with high mito count
```

```
# Assign cluster with >200 cells to cell lines (except cluster 2)
cellLine <- rep("uncertain", 19)
cellLine[7] <- "HEK"  # 1835 cells
cellLine[9] <- "THP1" # 1615 cells
cellLine[6] <- "KG1"  # 1157 cells
cellLine[5] <- "K562" #  999 cells
cellLine[8] <- "K562" #  808 cells
cellLine[3] <- "KG1"  #  387 cells
cellLine[11] <- "KG1" #  246 cells
sce$cellLine <- cellLine[cluster]
plotTSNE(sce, colour_by="cellLine") # Fig. 2A in manuscript

# Verify cluster assignment
boxplot(colSums(assay(altExp(sce))[c("HEK_A", "HEK_B", "HEK_C"), ]) ~ sce$cellLine)
boxplot(colSums(assay(altExp(sce))[c("THP1_A", "THP1_B", "THP1_C"), ]) ~ sce$cellLine)
boxplot(colSums(assay(altExp(sce))[c("K562_A", "K562_B", "K562_C"), ]) ~ sce$cellLine)
boxplot(colSums(assay(altExp(sce))[c("KG1_A", "KG1_B", "KG1_C"), ]) ~ sce$cellLine)
```
